# A novel heterozygous pathogenic *AIRE* variant causing autoimmunity but not infectious susceptibility

**DOI:** 10.70962/jhi.20250151

**Published:** 2025-10-09

**Authors:** Mounavi Vemula, Bergithe E. Oftedal, Dorsa Iraji, Mélanie Migaud, Christopher Richmond, Syndia Lazarus, Jean-Laurent Casanova, Anna Sullivan, Anne Puel, Stuart G. Tangye, Alberto Pinzon-Charry

**Affiliations:** 1 Queensland Paediatric Immunology & Allergy Service, Queensland Children’s Hospital, South Brisbane, Australia; 2 Department of Clinical Science, University of Bergen, Bergen, Norway; 3 St. Giles Laboratory of Human Genetics of Infectious Diseases, Rockefeller Branch, Rockefeller University, New York, NY, USA; 4 Laboratory of Human Genetics of Infectious Diseases, Necker Branch, INSERM U1163, Paris, France; 5 Imagine Institute, Paris Cité University, Paris, France; 6 Howard Hughes Medical Institute, New York, NY, USA; 7 Department of Pediatrics, Necker Hospital for Sick Children, Paris, France; 8 Genetic Health Queensland, Royal Brisbane and Women’s Hospital, Herston, Australia; 9 School of Medicine, Griffith University, Gold Coast, Australia; 10 Department of Diabetes and Endocrinology, Royal Brisbane and Women’s Hospital, Herston, Australia; 11 Clinical Immunogenomics Research Consortium Australasia (CIRCA), Darlinghurst, Australia; 12 Garvan Institute of Medical Research, Darlinghurst, Australia; 13 School of Clinical Medicine, Faculty of Medicine and Health, UNSW Sydney, Sydney, Australia; 14 Griffith University, Nathan Campus, Nathan, Australia; 15 University of Queensland, St Lucia, Australia

## Abstract

Autoimmune polyendocrinopathy-candidiasis-ectodermal dystrophy (APECED) is characterized by the triad of hypoparathyroidism, Addison’s disease, and chronic mucocutaneous candidiasis due to biallelic deleterious variants in *AIRE*. However, emerging evidence has established that some monoallelic variants affecting specific functional domains may also drive autoimmunity by negative dominance. Here, we describe a novel heterozygous *AIRE* variant, c.1010G>T (p.Cys337Phe), in three individuals from a Taiwanese-Singaporean family presenting with hypoparathyroidism, vitiligo, anemia, and ectodermal abnormalities, but not candidiasis. Functional studies confirmed AIRE^C337F^ is both loss-of-function and dominant negative to wild-type AIRE. Detection of neutralizing autoantibodies against type I IFNs, but not Th17 cytokines, further supported an APECED-like immunological profile and potentially explained the lack of infections in affected individuals. Like other dominant negative *AIRE* variants, AIRE^C337F^ localizes to the highly conserved PHD1 domain. Thus, our findings identify a novel pathogenic heterozygous *AIRE* variant and broaden the phenotype of autosomal dominant APECED. We also highlight the importance of functional validation in interpreting variants of unknown significance, particularly when disease prevalence and variant profiles differ from typical cohorts.

## Teaser

We describe a novel heterozygous variant in AIRE in 3 individuals with mild APECED. This variant was validated as being pathogenic by a mechanism of negative dominance. This represents new cases of autosomal dominant APECED.

## Introduction

Biallelic loss-of-expression or loss-of-function (LOF) variants in the autoimmune regulator (*AIRE*) gene form the genetic basis of autosomal recessive (AR) autoimmune polyendocrinopathy-candidiasis-ectodermal dystrophy syndrome (APECED), also known as autoimmune polyendocrine syndrome type 1 (1, 2, 3). *AIRE* plays a vital role in central immune tolerance by inducing expression of tissue-specific antigens (TSA) in the thymus, leading to elimination of autoreactive T cells and induction of T regulatory cells (4). Patients with APECED develop organ-specific autoimmune endocrinopathies—most commonly hypoparathyroidism and primary adrenal insufficiency, chronic mucocutaneous candidiasis (CMC), and neutralizing autoantibodies (autoAbs) against a range of cytokines, including type 1 IFNs and Th17-associated cytokines (4, 5, 6, 7, 8). Secondary manifestations, such as gonadal failure, thyroid disease, type 1 diabetes mellitus, vitiligo, enamel hypoplasia, alopecia, keratitis, and autoimmune liver disease may also be observed (4, 6, 9). The clinical presentation can be highly variable even among sibling pairs, complicating precise assertions of genotype–phenotype correlations (4, 6, 9). The spectrum of disease heterogeneity likely results from the differential impact of >100 identified *AIRE* variants on protein function, as well as epigenetic and environmental influences (10). Epidemiologically, APECED occurs with higher incidence in European populations such as Sardinians, Finns, and Iranian Jews (1:9,000–1:25,000) (11, 12); however, incidence among Asian populations is less well described (1:10 million among Japanese population) (13, 14, 15).

While classic APECED is caused by biallelic LOF variants in *AIRE* (1, 2, 4, 10), previous studies have identified monoallelic pathogenic variants in exons encoding the Sp100, AIRE, NucP41/75, and DEAF-1 (SAND); plant homeodomain 1 (PHD1); and PHD2 domains that exert a dominant-negative (DN) effect on wild-type (WT) AIRE protein (10, 16, 17, 18, 19, 20). These heterozygous variants result in a milder clinical phenotype than AR APECED and can have delayed onset and incomplete penetrance (10, 16, 17, 18, 19, 20). Affected individuals may also exhibit a broad clinical spectrum, ranging from an absence of overt autoimmune symptoms to severe enteropathy, vitiligo, immunodeficiency, and the variable presence of anti-cytokine and other autoAbs (10, 16, 17, 18, 19, 20).

Expanding on these prior findings, we describe and functionally characterize a novel heterozygous *AIRE* variant, c.1010G>T, p.(Cys337Phe [C337F]), affecting the PHD1 domain in three individuals across two generations in an Australian family of Taiwanese-Singaporean descent. Affected individuals presented with a dominantly inherited phenotype of ectodermal dysplasia and autoimmunity, but without CMC. The AIRE C337F variant was demonstrated to be LOF in terms of inducing transcription of *AIRE*-regulated genes and inhibited gene induction by WT AIRE protein. Thus, our findings further reveal the genetic diversity underlying APECED due to monoallelic variants and negative dominance.

## Results

### A novel heterozygous *AIRE* variant (p.Cys337Phe) identified in a family with clinical features of mild APECED

We describe three members of one family with a limited spectrum of APECED, including autoimmune polyendocrinopathy and ectodermal dystrophy, but without candidiasis. The 9-year-old male proband (II:1; Fig. 1) was referred to the pediatric immunology clinic with a history of pernicious anemia, hypoparathyroidism, and hyperglycemia. He was born to non-consanguineous parents of Taiwanese (maternal) and Singaporean (paternal) descent. His past medical history was notable for recurrent febrile seizures, persistent hypocalcaemia with nephrocalcinosis, chronic iron deficiency anemia, recurrent aphthous stomatitis associated with pharyngitis, and constipation. He also exhibited ectodermal abnormalities including enamel hypoplasia, hypodontia, and brittle nails, and later developed vitiligo. Of relevance, the patient’s younger sister (II:2) was already known to the immunology service for chronic spontaneous urticaria and hypoparathyroidism, with symptom onset at 4 years of age. Both children displayed normal anthropometry and had no additional morphological features. Immunological investigations, including serum immunoglobulin levels, lymphocyte subsets, and memory B cell phenotyping, were within age-matched reference ranges. Lymphocyte proliferation assays showed intact responses to phytohemagglutinin, but absent responses to candida antigens in both siblings. Celiac disease screen was negative, and serial assessments of glycated hemoglobin (HbA1C), thyroid function, cortisol, and adrenocorticotropic hormone levels have remained normal.

**Figure 1.**
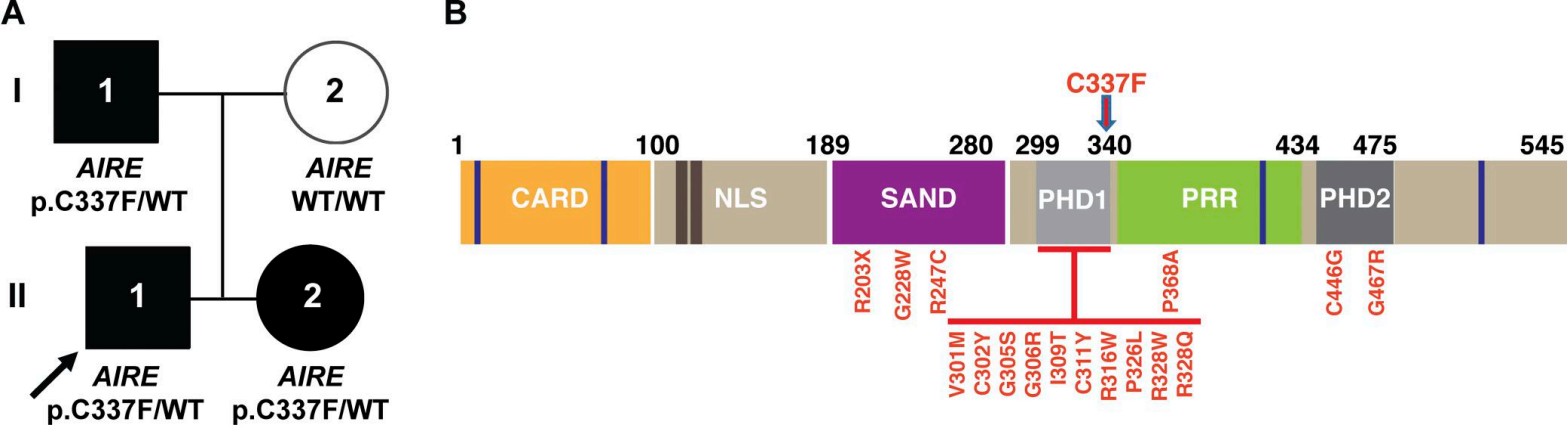
**Autosomal dominant AIRE deficiency. (A)** Pedigree of a family with a DN LOF *AIRE* variant. The arrow denotes the proband (II:1). **(B)** Schematic of AIRE protein depicting the different functional domains and as well as *AIRE* variants/amino acid substitutions affecting the SAND, PHD1, and PHD2 domains that have been previously identified in individuals with autosomal dominant APECED. The novel variant found in the family under investigation in our study is indicated by the red arrow (C337F) (see refs 16, 17, 18, 19, 20).

Family history revealed that the children’s father (I:1) had developed symptoms of hypoparathyroidism at the age of 10 years, characterized by muscle cramps, severe tetany, and profound hypocalcaemia requiring treatment with calcium, vitamin D, and hydrochlorothiazide. He had impaired fasting glucose, and his annual HbA1c was in the prediabetic range. There was no reported history of CMC in the proband, his sister, or their father.

Gene panel testing in the proband (II:1), his sister (II:2), and father (I:1) identified a heterozygous missense variant in *AIRE* (c.1010G>T, p.C337F) in all three individuals, confirming paternal inheritance and co-segregation with the clinical phenotype. The panel included 14 genes associated with hypo- or hyperparathyroidism, including *AIRE*, *AP2S1*, *CASR*, *CDC73*, *CDKN1A*, *CDKN1B*, *CDKN2B*, *CDKN2C*, *GCM2*, *GNA11*, *MEN1*, *PTH*, *RET*, and *TRPV6*. The AIRE^C337F^ variant is absent in the Genome Aggregation Database and has not previously been reported in other disease-associated variant databases (21). In silico predictions were deleterious (AlphaMissense score: 0.97, “likely pathogenic”), and the predicted substitution occurred within the conserved PHD1 of the protein. PHD1 is a zinc-coordinated domain critical for chromatin interaction and recognition of posttranslational histone modifications (22, 23). The clinical genetic laboratory classified the finding as a variant of uncertain significance (VUS). Fig. 1 B depicts the AIRE protein, including the different functional domains; the location of the novel variant introducing the C337F substitution in the PHD1 domain of AIRE, as detected in the proband (II:1), his sister (II:2), and father (I:1); and all amino acid substitutions resulting from monoallelic *AIRE* variants that have been previously identified in affected individuals and established as causal for AD APECED, are also shown (10, 16, 17, 18, 19, 20).

### Cytokine and organ-specific autoAb screening

**Figure 2.**
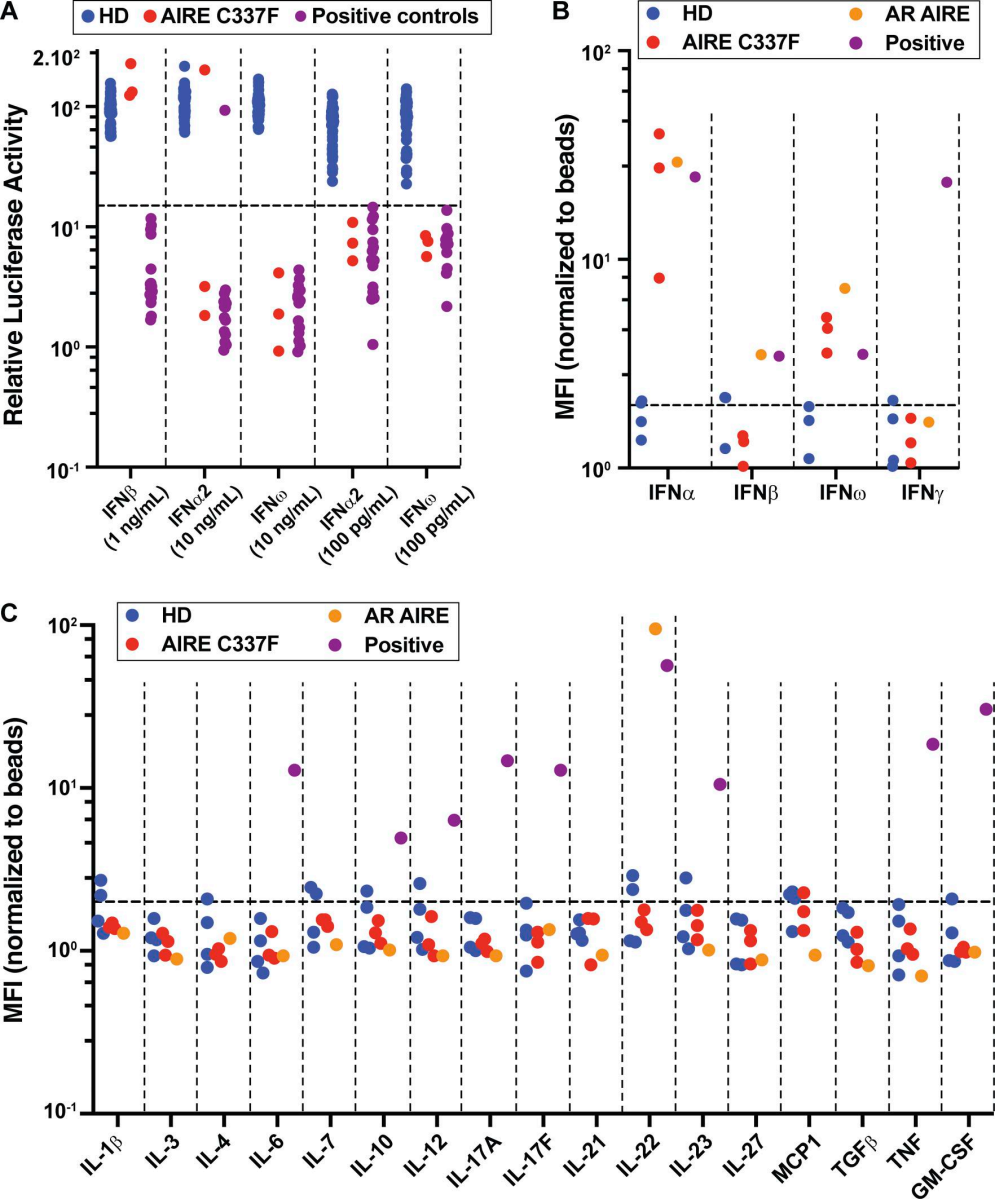
**Detection of autoAbs against type I IFNs in AIRE** ^**C337F**^ **individuals. (A)** Neutralization of type I IFNs determined by a Renilla luciferase reporter assay using transfected HEK-293T cells treated with IFN-α2 (10 ng/ml or 100 pg/ml), IFN-β (10 ng/ml), or IFN-ω (10 ng/ml or 100 pg/ml) in the absence or presence of plasma from healthy donors (HD) (*n* = 48, blue circles), individuals with the heterozygous *AIRE*^C337F^ variant (*n* = 3, red circles), or patients with autoAbs against type I IFNs (*n* = 15, purple circles; positive controls). Data are expressed as relative luciferase activity (ISRE dual luciferase activity, with normalization against Renilla luciferase activity) (24, 25). **(B and C)** Multiplex particle-based assay to detect autoAbs against (B) type I IFNs (IFNα2, IFNβ, and IFNω) and IFNγ or (C) IL-1, IL-3, IL-4, IL-6, IL-7, IL-10, IL-12, IL-17A, IL-17F, IL-21, IL-22, IL-23, IL-27, MCP-1, TGF β, TNF, and GM-CSF present in plasma from healthy donors (*n* = 4, blue circles), individuals with the heterozygous *AIRE*^C337F^ variant (*n* = 3, red circles), a patient with AR APECED (orange circles), or serum samples obtained from individuals with autoAbs against IFNα2, IFNβ, IFNω, IFNγ, IL-6, IL-10, IL-12, IL-17A, IL-17F, IL-22, IL-23, TNF, and GM-CSF (purple circles; positive controls). NB: Serum containing autoAbs against IL-β, IL-3, IL-4, IL-7, IL-21, IL-27, MCP-1, and TGFβ were not available for testing as positive controls at the time of testing the AD AIRE-deficient individuals (24, 25).

Given the strong clinical phenotype and segregation of the AIRE^C337F^ variant in all affected individuals, further investigation into its potential functional significance was explored. Neutralizing autoAbs against type 1 IFNs (IFN-α, IFN-β, and IFN-ω) and Th17 cytokines are a hallmark of AR APECED (4, 5, 7, 8). Consequently, we tested serum from the three affected individuals for anti-cytokine autoAbs (24). Sera collected from the proband (II:1) and his father (I:1) strongly reduced IFN signaling induced by both high (10 ng/ml) and low (100 pg/ml) concentrations of IFN-α and IFN-ω (Fig. 2 A and Table 1), demonstrating the presence of neutralizing autoAbs against type I IFNs. The proband’s sister, II:2, also showed neutralizing autoAbs against high and low concentrations of IFN-ω, but only against low concentrations of IFN-α (Fig. 2 A and Table 1). None of the affected individuals displayed evidence of neutralizing autoAb activity against IFN-β (Fig. 2 A and Table 1). We extended these findings by measuring autoAbs against type I (IFN-α, IFN-β, and IFN-ω) and type II (IFN-γ) IFNs using Multiplex bead arrays (24, 25). This assay detected autoAbs binding to IFN-α and IFN-ω, but not IFN-β or IFN-γ, in serum from all three affected individuals (Fig. 2 B). AutoAbs against IL-1, IL-3, IL-4, IL-6, IL-7, IL-10, IL-12, IL-17A, IL-17F, IL-21, IL-22, IL-23, IL-27, MCP-1, TGFβ, TNF, or GM-CSF were not detected in serum collected from any of the affected individuals (Fig. 2 C and Table 1).

**Table 1.** Clinical and serological features of family members with a heterozygous AIRE^C337F^ variant

Patient	Sex/Age	Clinical manifestations	autoAbs					
			IFN-α	IFN-ω	IFN-β	IL-6, IL-17A, IL-17F, IL-22	TNF, GM-CSF	Organ-specific
P1 II:1	M (9 yo)	HP, PA, V, and ED	Pos	Pos	Neg	Neg	Neg	ICA, GAD, and IF
P2 II:2	F (7 yo)	HP, CSU, and ED	Pos	Pos	Neg	Neg	Neg	-
P3 I:1	M (43 yo)	HP and PD	Pos	Pos	Neg	Neg	Neg	GAD

yo, years old; HP, hypoparathyroidism; PD, prediabetes; PA, pernicious anemia; V, vitiligo; ED, enamel dysplasia; CSU, chronic spontaneous urticaria; GAD, glutamic acid decarboxylase autoAbs; ICA, islet cell autoAb; IF, intrinsic factor autoAbs.

Organ-specific autoAb screening revealed positivity for glutamic acid decarboxylase Abs in individuals I:1 and II:1, with positivity for islet cell and intrinsic factor Abs observed exclusively in patient II:1. Abs against thyroid peroxidase, thyroglobulin, anti-tissue transglutaminase, IA-2, and zinc transporter 8 were negative in both siblings. Patient II:2 tested negative for all organ-specific autoAb evaluated, as summarized in Table 1.

### Dominant negative effect of C337F on induction of AIRE-dependent gene expression

**Figure 3.**
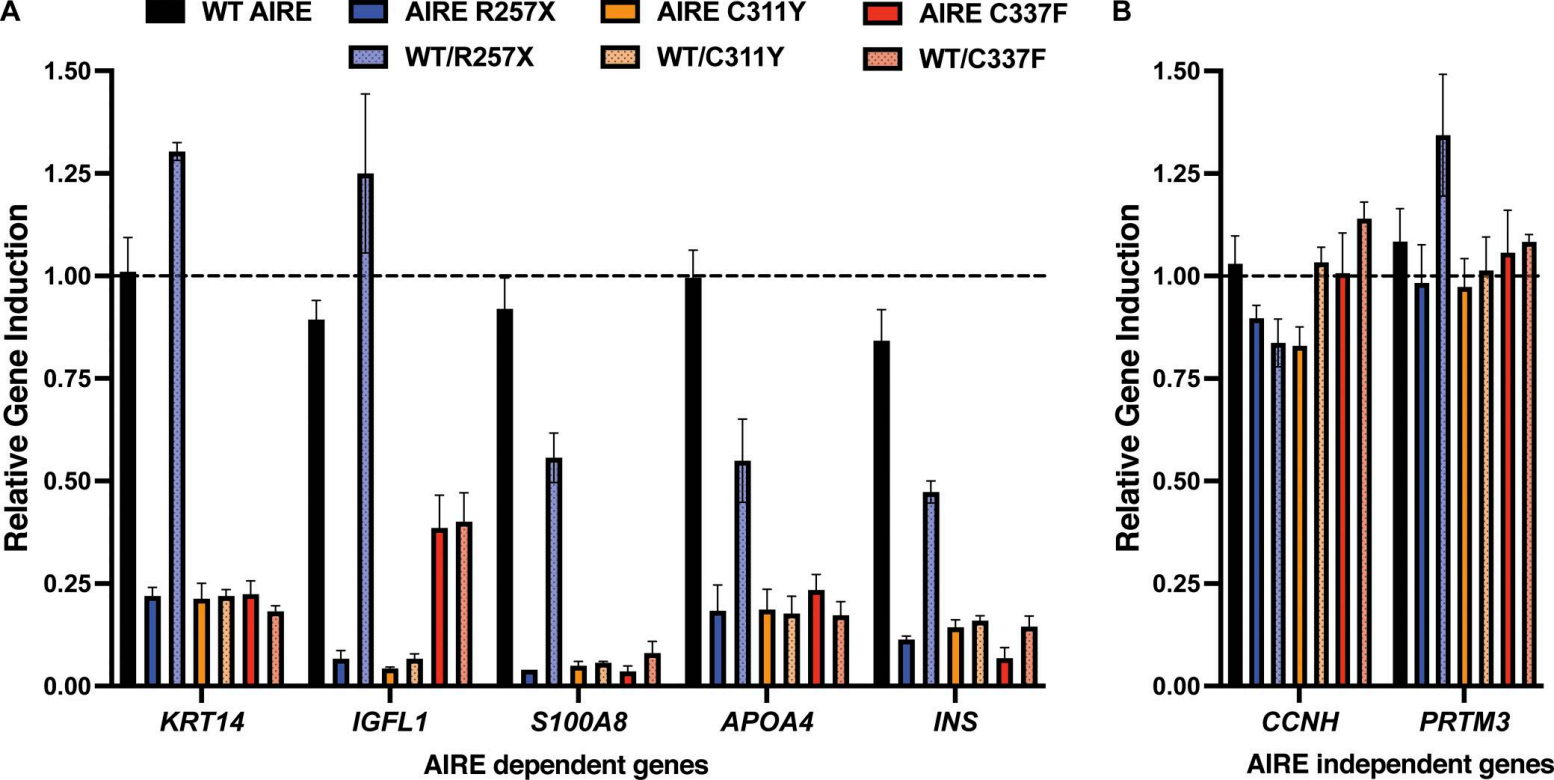
**DN impact of AIRE** ^**C337F**^ **on WT AIRE-mediated gene transcription.** HEK-293T cell lines were transfected with plasmids encoding either WT *AIRE* or AIRE^R257X^, AIRE^C311Y^ or AIRE^C337F^ variants alone, or equal amounts WT AIRE together with AIRE^R257X^, AIRE^C311Y^, or AIRE^C337F^. **(A and B)** Transcriptional activity was assessed by measuring expression levels of (A) known *AIRE*-regulated genes *KRT14*, *IGFL1*, *S100A8*, *APOA4*, and *INS* or (B) non-*AIRE*-regulated genes *CCHN* and *PRMT3*. Data are presented as mean fold-change in expression relative to cells transfected with WT 100% used as the calibrator sample (dotted line). Error bars represent the standard error of the mean from three independent experiments.

We next determined the impact of the c.1010G>T/p.C337F variant on induction of AIRE target genes. To do this, HEK-293T cells were transfected with expression vectors encoding either WT AIRE alone or AIRE^C337F^ alone. We also tested two other *AIRE* variants that are known to be pathogenic when biallelic (AIRE^R257X^) or monoallelic (AIRE^C311Y^) (16, 17, 18) As expected, WT AIRE robustly induced transcription of well-established AIRE-dependent target genes, including *keratin 14* (*KRT14*), *IGF-like family member 1* (*IGFL1*), *calcium-binding protein A8* (*S100A8*), *apolipoprotein A4* (*APOA4*), and *insulin *(*INS*) (Fig. 3 A). In contrast, transfection with AIRE^R257X^ or AIRE^C311Y^ failed to induce mRNA expression of any of these genes, while AIRE^C337F^ alone resulted in greatly reduced expression of *KRT14*, *S100A8*, *APOA4*, and *INS* and modestly reduced levels of *IGFL1* (Fig. 3 A). These results indicate that AIRE^C337F^ encoded by the novel *AIRE* variant is LOF, similar to AIRE^C311Y^, which is also located within the PHD1 domain of AIRE protein.

We further examined whether the AIRE^C337F^ variant could interfere with the transcriptional activity of WT AIRE, as has been reported in other individuals with milder forms of APECED and heterozygous *AIRE* variants, including the AIRE^C311Y^ variant (16, 17, 18). Co-transfection of HEK-293T cells with a 1:1 mixture of both *AIRE*^WT^ and *AIRE*^C337F^ vectors resulted in a level of transcription of AIRE-target genes comparable to that observed with AIRE^C337F^ alone (Fig. 3 A). Similar results were obtained for AIRE^C311Y^ (Fig. 3 A), confirming that the c.1010G>T/p.C337F variant impedes the function of WT *AIRE* by a mechanism of negative dominance. In contrast, the *AIRE*^C257X^ variant had either no effect (*KRT14* and *IGFL1*) or a less than twofold effect (*s100A8*, *APOA4*, and *INS*) on the ability of *AIRE*^WT^ to induce expression of AIRE-dependent target genes (Fig. 3 A), thereby establishing that the *AIRE*^C257X^ variant is strongly pathogenic only in homozygous form.

Importantly, induction of *AIRE*-independent genes, including *cyclin H* (*CCHN*) and *protein arginine methyltransferase 3* (*PRMT3*), was unaffected across all conditions (Fig. 3 B). Collectively, these findings demonstrate that AIRE^C337F^ is LOF and exerts a DN effect on WT AIRE-mediated transcriptional regulation in vitro. The selective loss of AIRE target gene expression, with preservation of AIRE-independent gene regulation, supports a specific disruption of canonical AIRE-mediated transcription. These findings suggest that AIRE^C337F^ is likely to disrupt central immune tolerance in a manner analogous to that seen in biallelic AIRE deficiency, supporting its pathogenic role in nonclassical APECED-like disease.

## Discussion

In this study, we describe a novel heterozygous deleterious *AIRE* variant c.1010G>T/p.C337F in three family members with similar clinical features, including autoimmune polyendocrinopathy and ectodermal dystrophy without CMC, and provide functional evidence for LOF and negative dominance of this variant allele. Our findings are consistent with previous reports that identified monoallelic variants affecting the PHD1 domain that impair expression of AIRE-regulated genes induced by WT AIRE.

The immune phenotype observed in this family overlaps with that reported in AR APECED, including the presence of neutralizing autoAbs to type I IFN (IFNα and IFNω), which are recognized as a hallmark of classical APECED (4, 5, 7, 8). Notably, autoAbs against IL-17A, IL-17F, and family IL-22 were not detected in the affected individuals. Thus, the lack of these autoAbs is consistent with an absence of CMC in this family, which is frequently observed in classical AR APECED and has been attributed to impaired IL-17-mediated immunity to *Candida albicans* (7, 8, 10, 26, 27, 28). IFN-γ–mediated mucosal inflammation has recently been implicated as another mechanism underlying CMC in AR APECED (29). DN *AIRE* variants resulting in milder phenotypes may confer a reduced propensity for developing significant mucosal inflammation (10, 16, 17, 18, 19, 20). Although CMC is uncommon in AD APECED, a few reported patients with heterozygous *AIRE* variants that affect the SAND or PHD1 domains have developed fungal infection/mycosis in the absence of detectable anti-IL-17 autoAbs (∼10–15% vs. >90% in AR APECED) (10, 16, 17, 18, 19, 20). This observation suggests that additional mutation-specific, environmental, and immunological factors may modulate mucosal susceptibility to CMC in the context of heterozygous *AIRE* variants.

DN *AIRE* variants are largely clustered within the PHD1 zinc finger domain, with some variants also affecting the SAND and PHD2 domains (Fig. 1 B). The clinical phenotype associated with these monoallelic variants ranges from asymptomatic to autoimmunity (commonly vitiligo and enteropathy) and immune deficiency, with or without production of autoAbs neutralizing type 1 IFNs (10, 16, 17, 18, 19, 20). However, cases due to monoallelic *AIRE* variants like the ones described here often present with milder disease and incomplete penetrance (10). These are collectively referred to as “nonclassical APECED,” which differ from the classical form caused by biallelic deleterious *AIRE* mutations characterized by earlier onset CMC, hypoparathyroidism, or adrenal insufficiency (16). The proband in our study presented with vitiligo and pernicious anemia, which have been previously linked to DN *AIRE* variants affecting the PHD1 domain (16, 18). However, hypoparathyroidism as an early feature in all three affected family members (onset between 4 and 10 years) suggests a more pronounced and earlier onset autoimmune phenotype than commonly reported for monoallelic *AIRE* variants affecting the PHD1 domain (10, 16, 17, 18, 19, 20). This observation highlights the clinical variability even among individuals with monoallelic *AIRE* variants.

Variants affecting the caspase activation and recruitment domains (CARD) domain of AIRE are disease-causing when inherited as an autosomal recessive trait, impairing nuclear localization and interfering with oligomerization, thereby disrupting the ability of *AIRE* to dimerize and activate transcription (30). However, when co-expressed with WT *AIRE*, these CARD domain mutants may still permit formation of functional dimers, thus explaining why heterozygous carriers of variants that are only pathogenic in biallelic form do not develop disease (16). Our findings suggest that the AIRE^C337F^ variant affects the PHD1 domain, resulting in a clear DN effect on WT AIRE function. This is consistent with previous data that stipulate monoallelic variants within the PHD1 domain disrupt the structural integrity of the core AIRE tetramer and its transcriptional activity (16, 31). By enabling AIRE to bind unmethylated H3K4 and promote TSA expression in medullary thymic epithelial cells, the PHD1 domain of AIRE is critical for central immune tolerance (32, 33, 34). Mutations at conserved cysteine residues within this domain, such as C311Y, disrupt zinc coordination, leading to impaired domain folding and function (16, 31, 35).

In conclusion, by characterizing the novel p. C337F variant affecting the PHD1 domain, our report expands the phenotypic and spectrum of DN *AIRE* variants. Functional characterization of VUS remains critical for determining their causal role in nonclassical presentations of APECED. These findings emphasize that, similar to classical APECED, nonclassical forms of the disease can also exhibit marked phenotypic heterogeneity and intrafamilial variability, even among sibling pairs.

## Materials and methods

### Research subjects

The patients were recruited following identification of a unique *AIRE* variant in 2023–2025 at the Queensland Children’s Hospital. This study was approved by the Sydney Local Health District Royal Prince Alfred Hospital Zone Human Research Ethics Committee and Research Governance Office, Royal Prince Alfred Hospital, Camperdown, Australia (Protocols X16-0210/LNR/16/RPAH/257 and X16-0210 and 2019/ETH06359, and Protocol X20-0177 and 2020/ETH00998). Informed consent for functional studies was obtained from the family. Permission for publication was obtained from the family and Children’s Health Queensland Hospital and Health Service Human Research Ethics Committee.

### *AIRE* gene sequencing

Custom gene panel testing in I:1 was performed in a clinical laboratory improvement amendments (CLIA)- and college of American pathologists (CAP)-accredited laboratory (Blueprint Genetics) on DNA extracted from peripheral blood, analyzing 14 hyper-/hypoparathyroidism-associated genes (*AIRE*, *AP2S1*, *CASR*, *CDC73*, *CDKN1A*, *CDKN1B*, *CDKN2B*, *CDKN2C*, *GCM2*, *GNA11*, *MEN1*, *PTH*, *RET*, and *TRPV6*) for sequence and small copy number variants. Targeted gene panel was performed using a targeted sequence capture method followed by next-generation sequencing of the amplified captured regions (Illumina). Alignment to reference genome GRCh37 was performed, and annotated variants were classified according to modified American College of Medical Genetics and Genomics and the Association for Molecular Pathology guidelines (36). Copy number analysis revealed no deletions or duplications at the exon level within *AIRE*.

### AutoAb analysis

Abs to tissue transglutaminase were assessed using Chemiluminescence (Werfen), and Abs to islet cell, thyroid peroxidase and thyroglobulin, anti-tissue transglutaminase, glutamic acid decarboxylase, IA-2, intrinsic factor, and zinc transporter 8 were assessed using enzyme-linked immunosorbent assays (Fadia and Abacus diagnostics). The diagnosis of endocrinopathies was established using laboratory results and clinical features as previously described (3, 10, 11).

### Detection of neutralizing autoAbs against type I IFNs

Neutralizing autoAbs against type I IFNs were detected in serum of individuals with heterozygous *AIRE* variants using a previously described luciferase assay (25). HEK-293T cells were transfected with a plasmid containing the firefly luciferase gene under the control of the human *ISRE* promoter; the cells were preincubated with serum 10% from healthy donors or individuals with heterozygous *AIRE* variants and then treated with different amounts of type I IFNs. After 16 h, cells were lysed, and luciferase levels were measured with the Dual-Luciferase Reporter 1000 Assay System (25).

### Detection of anti-cytokine autoAbs by multiplex particle-based assay

AutoAbs to IFNα, IFNβ1, IFNω, IFN-γ, IL-4, IL-6, IL-7, IL-10, IL-12, IL-17A, IL-17F, IL-21, IL-22, IL-23, IL-27, MCP-1, TGFβ, TNF, and GM-CSF were assessed using multiplex assay detection by flow cytometry (24, 25). BD Cytometric Bead Array (BD CBA Flex system) were coated with 10 μg of recombinant human cytokine (IFN-α, IFN-β, IFN-ω, IFN-γ, IL-12p40, IL-17A, IL-23, IL-6, and GM-CSF; Bio-Techne) according to the manufacturer’s instructions (558556; BD). After validation of the coupling, the beads were incubated for 2 h with serum from healthy donors, patients, or positive controls (1/1,000 dilution in PBS/2% BSA). After washing twice with PBS/0.005% Tween, beads were incubated with a PE goat anti-human IgG Ab (C3923-S083E; Southern Biotech). Two washes in PBS/0.005% Tween were then performed. Finally, the beads were acquired on an Agilent Novocyte NovoSampler Pro, and data were analyzed using the FlowJo software v.10.6.2 (Becton Dickinson) (24, 25).

### Cell transfection and AIRE-regulated gene assays

The plasmid svPoly containing human WT *AIRE* was a kind gift from Dr. Ismo Ulamanen (National Institute for Health and Welfare, Department of Molecular Medicine, Biomedicum, Helsinki, Finland). The C337F mutation was introduced by site-directed mutagenesis (QuickChange II Site-Directed Mutagenesis Kit, Agilent Technologies) using the following primers (5′-3′):

Forward: 5′-GGA​CCT​GGA​GGT​TCT​CCA​GCT​GCC​TG-3′;

Reverse: 5′-CAG​GCA​GCT​GGA​GAA​CCT​CCA​GGT​CC-3′, designed by the web-based program PrimerX (https://www.bioinformatics.org/primerx) and verified by Sanger sequencing.

HEK293 cells were grown in Dulbecco’s Modified Eagle Medium high glucose (Sigma-Aldrich) supplemented with 10% (vol/vol) fetal bovine serum, 10 mM HEPES buffer, 1% (vol/vol) nonessential amino acids (Lonza), 2 mM L-glutamine (Lonza), 100 U/ml penicillin, and 100 μg/ml streptomycin (Lonza) at 37°C with 5% CO_2_ in a humidified incubator. For transfection, cells were plated at a density of 5 × 105 cells per well in a 6-well plate and left in the humified incubator overnight. Samples (3.3 μg) of the svPoly plasmids were added to a total volume of 157 μl supplemented RPMI 1640 (without penicillin or streptomycin), mixed with 8.3 μl of Fugene HD transfection reagent (Promega Corporation), and incubated for 5 min at room temperature. After adding the mixture to the cells, they were incubated for 24 h before total RNA was extracted by RNeasy Mini Kit (QIAGEN) according to the manufacturers’ protocol, including in-column DNase treatment. cDNA was prepared from 1 μg of total RNA via a High Capacity RNA-to-cDNA Kit (Applied Biosystems). HEK293 cells were transfected with either WT *AIRE* (100% WT), mutant *AIRE* alone (100% mutant), or mixture of WT and mutant *AIRE* plasmids (50% mutant). In all assays a negative control (plasmid with no insert) was included.

Genes previously shown to be regulated by *AIRE* were analyzed by quantitative PCR using the following primers and probes (Applied Biosystems): *S100A8* (Hs0037444264_g1), *KRT14* (Hs00265033-m1), *IGFL1* (Hs01651089-g1), *APOA4* (Hs00166636_m1), and *INS* (Hs02741908_m1) (37, 38). Results were compared to *Beta2-microglobulin* (*B2M*) (4333766) as endogenous control, and the *AIRE*-independent genes *CCHN* (Hs00236923_m1) and *PRMT3* (Hs00411605_m1). Datasets of each primer pair were normalized to *B2M*. The fold difference was calculated as 2 − {Ct((target gene) − Ct(B2M)) − (Ct(test sample) − Ct(calibrator sample))}, with test samples defined as the different mutants of *AIRE* and calibrator as WT *AIRE*. The results are shown as the mean of three biological replicates, and results are expressed as mean ± SEM.

## Data Availability

The raw data supporting the conclusions of this article will be made available by the corresponding author(s) upon reasonable request.
